# Isotropic, aberration-corrected light sheet microscopy for rapid high-resolution imaging of cleared tissue

**DOI:** 10.1038/s41587-025-02882-8

**Published:** 2025-11-13

**Authors:** Mostafa Aakhte, Gesine F. Müller, Lennart Roos, Joe Li, Torben Göpel, Kurt R. Weiss, Aleyna M. Diniz, Jan Wenzel, Markus Schwaninger, Tobias Moser, Jan Huisken

**Affiliations:** 1 Multiscale Biology, Department of Biology and Psychology, University of Göttingen, Göttingen, Germany; 2 Institute for Auditory Neuroscience and InnerEarLab, University Medical Center Göttingen, Göttingen, Germany; 3 Else-Kröner-Fresenius Center for Optogenetic Therapies, University Medical Center Göttingen, Göttingen, Germany; 4 Department of Otorhinolaryngology, University Medical Center Göttingen, Göttingen, Germany; 5 Cluster of Excellence ‘Multiscale Bioimaging: from Molecular Machines to Networks of Excitable Cells’ (MBExC), University of Göttingen, Göttingen, Germany; 6 Morgridge Institute for Research, Madison, WI USA; 7 Department of Biochemistry, University of Wisconsin, Madison, WI USA; 8 Auditory Neuroscience and Synaptic Nanophysiology Group, Max Planck Institute for Multidisciplinary Sciences, Göttingen, Germany; 9 Center of Brain, Behavior and Metabolism, Institute of Experimental and Clinical Pharmacology and Toxicology, University of Lübeck, Lübeck, Germany; 10 German Research Centre for Cardiovascular Research (DZHK), Partner site Hamburg/Lübeck/Kiel and Göttingen, Göttingen, Germany

**Keywords:** 3-D reconstruction, Light-sheet microscopy, Fluorescence imaging, Neuroscience

## Abstract

Light-sheet microscopy is ideal for imaging large and cleared tissues, but achieving a high isotropic resolution for a centimeter-sized sample is limited by slow and often aberrated, axially scanned light sheets. Here, we introduce a compact, high-speed light-sheet fluorescence microscope achieving 850 nm isotropic resolution across cleared samples up to 1 cm³ and refractive indices ranging from 1.33 to 1.56. Using off-the-shelf optics, we combine an air objective and a meniscus lens with an axially swept light sheet to achieve diffraction-limited resolution and aberration correction. The effective field of view is increased by twofold by correcting the field curvature of the light sheet using a concave mirror in the remote focusing unit. Adapting the light sheet’s motion with a closed-loop feedback enhances the imaging speed by tenfold, reaching 100 frames per second while maintaining resolution and field of view. We benchmark the system performance across scales, from subcellular structure up to centimeter scale, using various clearing methods.

## Abstract (web summary)

A light-sheet microscope built from off-the-shelf components enables high-resolution clear tissue imaging.

## Main

Light-sheet fluorescence microscopy (LSFM) has become a key instrument in modern biology with its confined excitation and its capability to image live, intact biological samples fast and gently^1–4^. In LSFM, optical sectioning is achieved by two orthogonal optical arms^5^: one projects a laser light sheet into the sample, illuminating a thin section, and the other records the emitted fluorescence light perpendicularly with a camera. This configuration enables simultaneous collection of emitted light across the entire field of view (FOV), surpassing sequential point-scanning methods in photon efficiency and speed. Localized fluorescence excitation at the chosen plane greatly reduces photobleaching, which is critical for in vivo imaging, but LSFM is also an effective method for imaging large and cleared tissues^6–10^. It enables scientists to obtain high-resolution images across the entire intact sample, crucial for subsequent reconstruction and quantification.

The light-sheet properties, such as its thickness, effective length (Rayleigh length) and intensity distribution in all directions greatly influence image quality. The thickness of the light sheet primarily determines the axial resolution. In contrast, the lateral resolution is determined by the numerical aperture (NA) of the detection objective lens (DL), magnification and camera pixel size^5,11^. Consequently, the axial resolution can be enhanced by creating thinner light sheets. However, a thinner light sheet becomes shorter, no longer filling the entire FOV. Quantitative multiscale analysis requires the imaging setup to have an isotropic resolution, where the axial and lateral resolutions are the same across the entire FOV and, consequently, the illumination and detection NA need to be identical. Isotropic resolution across a large FOV can be achieved elegantly by rapidly moving a thin light sheet across the FOV using a remote-focusing optical arm—a technique known as axially swept light-sheet microscopy (ASLM)^12,13^. To record only the thinnest part of the light sheet across the FOV, the camera is oriented such that the rolling shutter direction aligns with the light sheet’s propagation direction (Supplementary Video 1). The light sheet is swept across the FOV once during the exposure time of the scientific complementary metal–oxide–semiconductor (sCMOS) camera, with its waist location synchronized to the position of the camera’s rolling shutter.

In ASLM, the light sheet can be swept with electrically tunable lenses (ETLs), piezo actuators or voice coil actuators. The concept was introduced initially using a piezo actuator for imaging small live samples, providing an isotropic resolution of 370 nm over a 50-μm FOV at an imaging rate of 6 Hz (ref. ^12^). ASLM was also adapted to larger cleared samples, as demonstrated in the MesoSPIM platform, which achieved a FOV of 13.29 mm with an isotropic resolution of 6.5 μm (ref. ^14^). The subsequent benchtop MesoSPIM variant offered an improved resolution, albeit nonisotropic, at 1.5 μm laterally and 3.3 μm axially, with frame rates of 4.5 Hz and 6.5 Hz using ETLs^15^. Another setup achieved isotropic resolution up to 380 nm, covering a FOV of 310 μm × 310 μm and using mechanical tiling to image large samples^13^. This setup employed a voice coil actuator, which provided higher stroke movement in continuous motion, increasing the frame rate to 10 Hz. However, the setups, so far, have not reached the maximum frame rate of the sCMOS camera. The speed, however, is crucial to keep acquisition times within reasonable limits, especially in large tissues where many tiles need to be acquired at high resolution to reconstruct the entire sample.

To support a wide range of applications, the microscope should be compatible with expansion microscopy^16,17^ and many tissue-clearing protocols such as 3DISCO, iDISCO, EZ Clear, and so on^18–20^ and their broad range of refractive indices (RIs), from RI = 1.33 (water) to RI = 1.56 (ethyl cinnamate; ECi). A straightforward approach to image samples with various RIs uses air objective lenses with long working distances^21,22^. Oily and toxic solvents are then enclosed in a chamber with glass windows, and the air lenses can be exchanged easily without touching the solvent. However, in such a configuration, the lenses induce substantial spherical, chromatic aberrations and field curvature, preventing reaching the diffraction limit. Spherical aberrations can be mitigated by introducing a meniscus lens instead of the flat glass window between the air objective lens and immersion medium^23^. An open-top light-sheet microscope^24,25^ used a customized meniscus lens to achieve a near-diffraction-limited light sheet. However, a low NA light sheet in combination with a high NA detection lens limited resolution to 1.1 μm laterally and 2 μm axially with an effective FOV of 780 μm × 375 μm because of aberrations at the edge of the detection arm.

Field curvature is also a common aberration, limiting the FOV and affecting both the detection and illumination arms. In the detection arm, field curvature results in blur at the edges of the image. On the illumination side, the waist of the light sheet is bent in the presence of field curvature, which is particularly pronounced for high NA. This effect leads to a desynchronization of the waist movement at the FOV edges in ASLM and reduces the usable FOV to a small area where the waist is straight. The field curvature can be addressed by reducing the FOV or designing an expensive custom objective lens^26^. One can also select lenses with minimum field curvature by measuring their field curvature^15,27^. Further, the existing field curvature can be adapted by adjusting the light-sheet geometry and using a curved beam^28^. Although all the above-mentioned efforts use at least one air objective lens with minimized aberrations, each approach falls short of achieving isotropic submicron resolution over the largest possible FOV.

To overcome all these issues, we have developed a compact, field curvature-corrected light-sheet microscope tailored for imaging cleared tissues. The microscope offers an isotropic resolution of 850 nm in media with RIs ranging from 1.33 to 1.56 at a speed of 100 frames per second to image samples up to 1 cm^3^ in size. Our innovative design combines a multi-immersion lens, an air objective lens, a meniscus lens and a concave mirror within an ASLM framework. We have doubled the FOV by using a concave mirror to correct the field curvature of the light sheet while maintaining isotropic resolution. Further, we have greatly improved the imaging speed by a factor of ten, compared to the fastest previous microscopes, by precisely controlling the ASLM unit at high frequencies with an optical closed loop. We benchmarked the system performance by imaging various biological samples, including cleared mouse brain, mouse cochlea and zebrafish.

## Results

We aimed to achieve an isotropic resolution <1 µm to image submicron structures in large, cleared samples in various media. Hence, the DL needed to have an NA of ≥0.4. Commercially available options included a multi-immersion lens (×16, NA 0.4, working distance around 12 mm, ASI), which achieves optimal sampling when paired with a 200 mm tube lens and an sCMOS camera with a typical pixel pitch of 6.5 μm (Supplementary Note 1 and Supplementary Fig. 1). This lens is already corrected for chromatic aberrations with constant working distance for different RIs, compatible with various media without the need to realign the lens when changing the medium, making it the ideal choice for the detection lens in our setup.

Conversely, an air objective lens with matching NA = 0.4 served as an ideal illumination lens. We placed it outside the interchangeable sample chamber and its toxic solvents. The air lens can be moved freely during alignment without requiring any sealing. The flexibility of the air illumination objective lens also enabled us to attach the detection lens in a fixed position, sealed to the chamber with a simple O-ring, and its focal plane in the chamber center aligned to the illumination’s optical axis. The illumination lens’ large working distance (20 mm in air) is beneficial for imaging large samples of a centimeter or more. The chosen Mitutoyo objective lens (×20 plan apochromat) has an NA of 0.42, which closely matches the detection lens’ NA—a prerequisite for achieving isotropic resolution. In addition, in ASLM, to minimize aberrations, the remote-focusing objective lens should ideally be identical to the illumination lens, which was easily achievable with this affordable, long working distance, air objective lens.

**Fig. 1:**
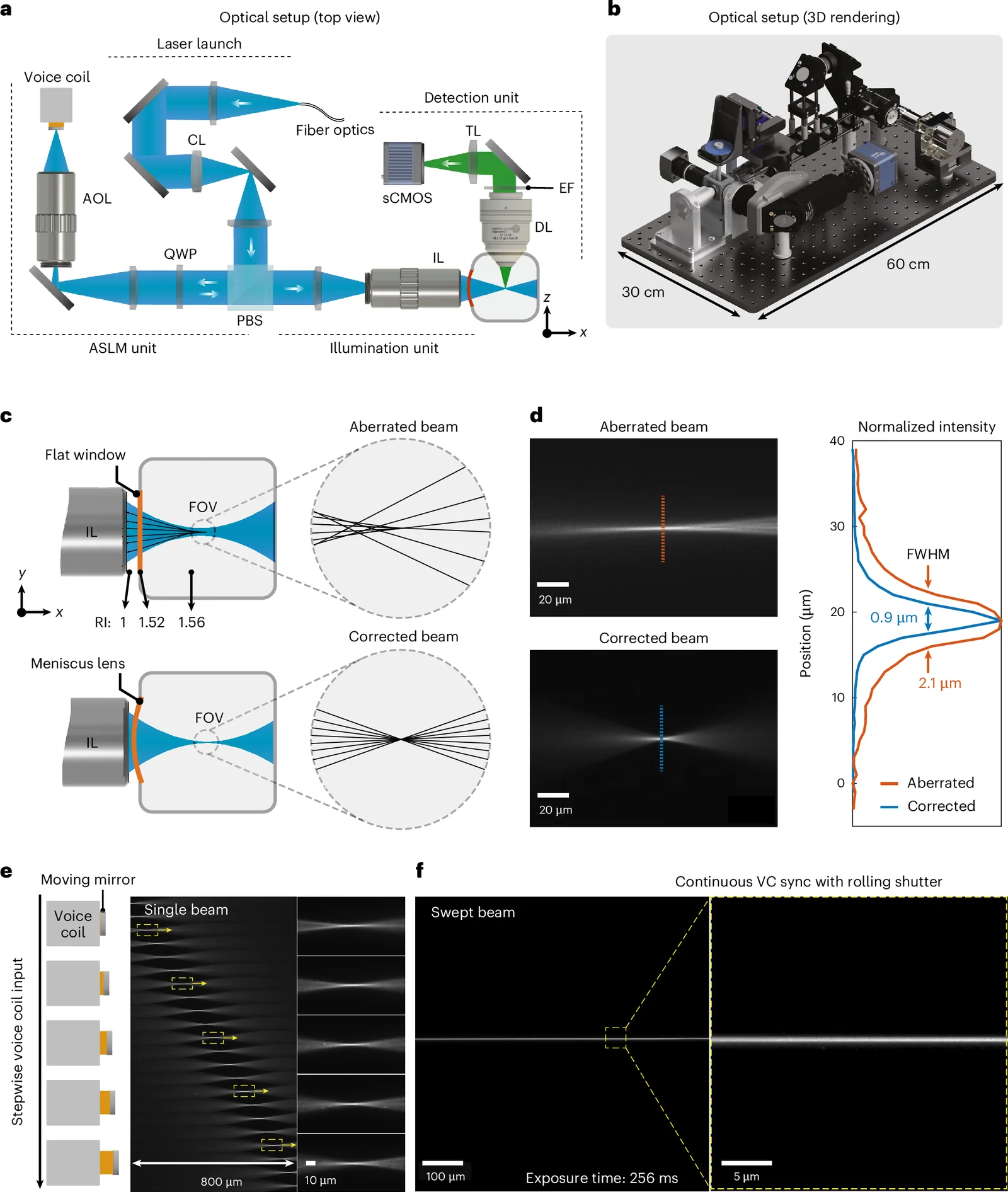
Axially swept cleared tissue light-sheet setup and diffraction-limited swept beam. **a**, Optical schematic of the cleared tissue setup from a top view. **b**, 3D rendering of the optical setup in **a**. **c**, Comparison of a glass window and a meniscus lens for correcting spherical aberration in the illumination beam. **d**, Single static beam in a chamber with a flat window and a meniscus lens. The graph shows the beam thickness for both cases. The images are examples from one of ten imaged beams. **e**, Schematic of the voice coil when its input voltage is changed stepwise. Second and third panels: recorded single beam across the detection FOV, demonstrating the absence of aberrations. **f**, Swept beam with the continuous motion of the voice coil synchronized with the sCMOS rolling shutter. The images are examples from one of five imaged beams. AOL, ASLM objective lens; IL, illumination objective lens; DL, detection objective lens; TL, tube lens; EF, emission filter; FWHM, full-width at half-maximum; PBS, polarizing beam splitter; QWP, quarter-wave plate.

Our microscope consists of four main optical components: the laser launch, the ASLM, the illumination arm and the detection arm (Fig. 1a,b, Supplementary Figs. 2–3 and Supplementary Table 2). Briefly, a laser beam with a Gaussian profile enters through the laser launch and is shaped into a light sheet using a cylindrical lens (CL). This light sheet then travels to the ASLM unit, where it is swept by a voice coil and its attached mirror, positioned opposite the ASLM objective lens. The voice coil acts as a single-axis translator, moving rapidly the mirror along the optical axis. Reflecting the primary light sheet through the ASLM and illumination arm results in a shifted light sheet within the chamber. The DL then collects the fluorescence emitted from the sample, and the image is formed by a tube lens on an sCMOS camera with a rolling shutter (Methods).

When using an air objective lens, one of the primary aberrations anticipated is spherical aberration, which prevents the light sheet from achieving the diffraction limit^23^ (Fig. 1c, first row). To mitigate spherical aberrations, we introduced an off-the-shelf meniscus lens between the air objective lens and the chamber instead of using a flat glass window. The meniscus lens has a curvature that matches the NA of the illumination beam to allow all the rays to enter the chamber perpendicular to the glass surface and to minimize refraction (Fig. 1c, second row). Comparing the beam quality, we saw strong spherical aberration when using the flat glass window, whereas it was eliminated greatly with the meniscus lens, reducing the beam size from 2.1 μm to 900 nm, the near-diffraction limit (Fig. 1d). We also examined the beam quality across the detection FOV to ensure it remained aberration-corrected when sweeping the light sheet. We achieved a homogeneous, confined beam across the entire FOV (800 μm × 800 μm) by combining ASLM with the meniscus lens. We then synchronized the swept aberration-corrected beam with the sCMOS rolling shutter during the exposure time, recording it over the FOV from left to right (Fig. 1f and Supplementary Video 2).

**Fig. 2:**
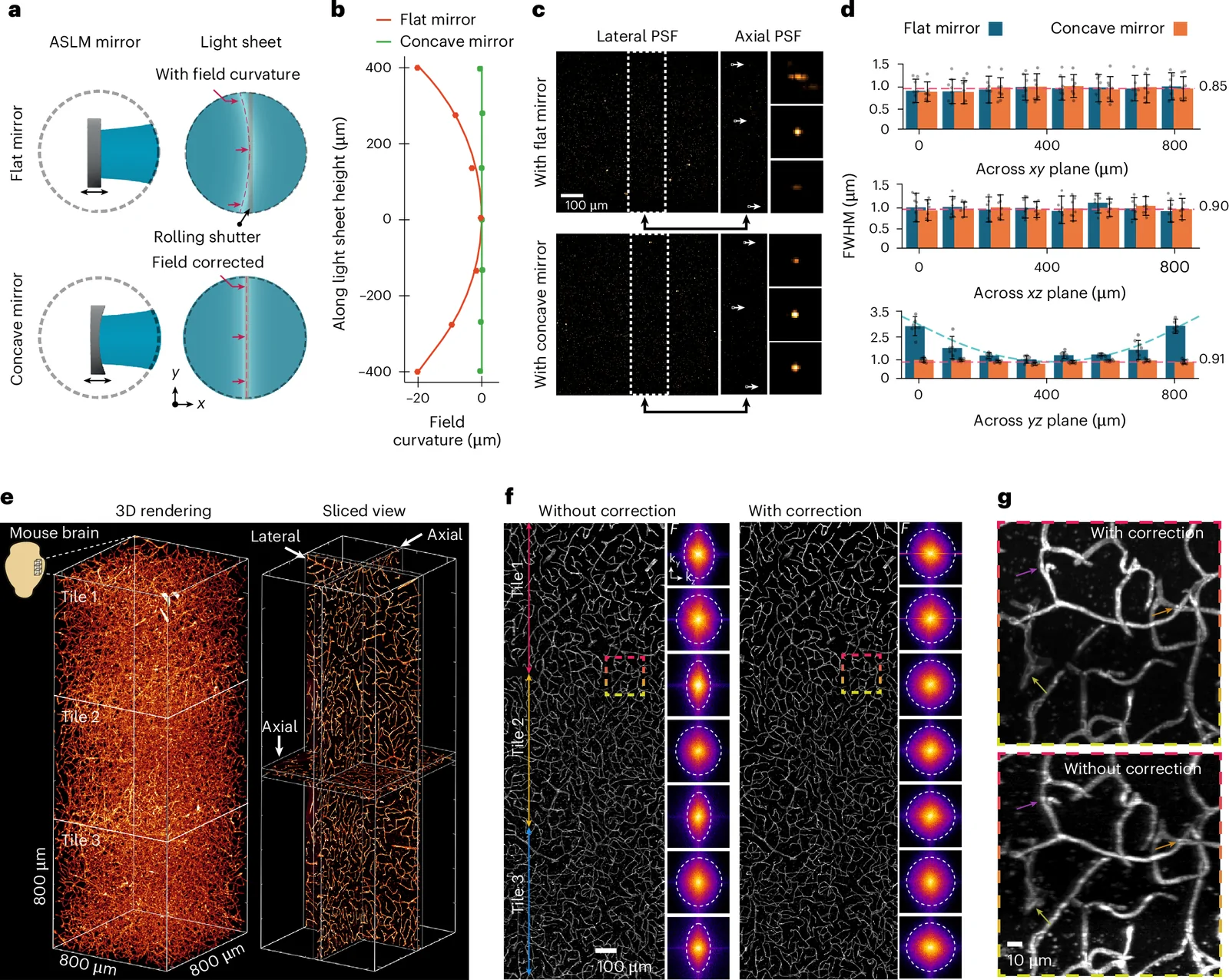
Flat-field-corrected light sheet enhances FOV and quality across the imaging tiles. **a**, Schematic comparison of using the flat mirror and curved mirror in the ASLM unit and their effect on the field curvature of the light sheet. **b**, Measured field curvature of the light sheet without (red) and with correction (green). **c**,**d**, PSF measurement with 200-nm nanoparticles and comparison without and with the field curvature correction in lateral and axial directions. The PSF FWHM (± s.e.m.) was measured for seven beads in each FOV section. **e**, 3D rendering and middle slices of the lateral and axial views of the recorded data from a cleared mouse brain, stained for microvessels with three tiles along the height of the light sheet. The images are examples from one of three imaged mouse brains. **f**, Axial view of the recorded data in **d** along the light-sheet height and the corresponding Fourier domain comparison with its associated cut of frequency for seven regions. **g**, Enlarged view of selected areas in **f** located between adjacent tiles. The arrowheads show the improvements in vessel information with field curvature correction.

Another essential requirement for ASLM is that the waist of the light sheet forms a straight line from top to bottom across the FOV. This is necessary to synchronize the thinnest part of the sweeping light sheet with the center of the rolling shutter. However, illumination field curvature is expected when using a high NA air objective lens in combination with high RI medium across a large FOV (Fig. 2a). To evaluate the illumination field curvature, we first imaged the light-sheet profile directly using a small mirror inside the chamber at 45 degrees between the optical arms. We then measured the center location of the light-sheet cross-section from top to bottom (Methods). The field curvature of the light sheet was 20 ± 0.2 μm with an excitation beam of 561 nm and RI of 1.56, which was nearly three times larger than the light-sheet Rayleigh length (Fig. 2b, red line). To correct the light-sheet field curvature, we explored replacing the conventional flat mirror in the ASLM unit with a concave mirror with an inverse curvature, as measured by the light-sheet field curvature. The chosen off-the-shelf concave mirror (CM127-010-P01, Thorlabs) has a 9.5-mm effective focal length with a maximum offset of 10 μm at 400 μm from its center, which covers the light sheet with an 800-μm height (Supplementary Fig. 4a). The field curvature was corrected to less than 1 μm along the light sheet (Fig. 2b, green line) compared to the flat mirror. Then, we characterized the axial resolution by imaging 200 nm nanoparticles and measuring the point spread function (PSF). When using the flat mirror, we measured a variety of axial PSFs over the light-sheet height from 800 nm to 2.5 μm (Fig. 2c). Here we achieved isotropic resolution only in the central 400 µm of the FOV, whereas the rest of the FOV remained nonisotropic. However, with the concave mirror, the axial resolution was homogeneous across the entire FOV, with an isotropic resolution of 850 nm. This enabled us to increase the effective FOV by a factor of two compared to the flat mirror (Fig. 2d).

To examine the microscope’s performance in cleared tissue, we used a mouse brain labeled with antibodies for brain endothelial cells, visualizing even the smallest vascular structures, so-called capillaries (Fig. 2e). The imaging was done for three tiles to evaluate the resolution across the volume, before and after field curvature correction (Fig. 2f). The axial view of the recorded images before field curvature correction showed that the cut-off frequency of the noncorrected images varied and decreased to almost half in the Fourier domain compared to the image with corrected field curvature. In contrast, image quality and the frequency distribution remained constant across the tiles when imaged with field curvature correction (Fig. 2g). Consequently, eliminating the light-sheet field curvature achieved isotropic resolution across a larger FOV and into the corners of the images, crucial for image stitching and quantitative imaging.

**Fig. 3:**
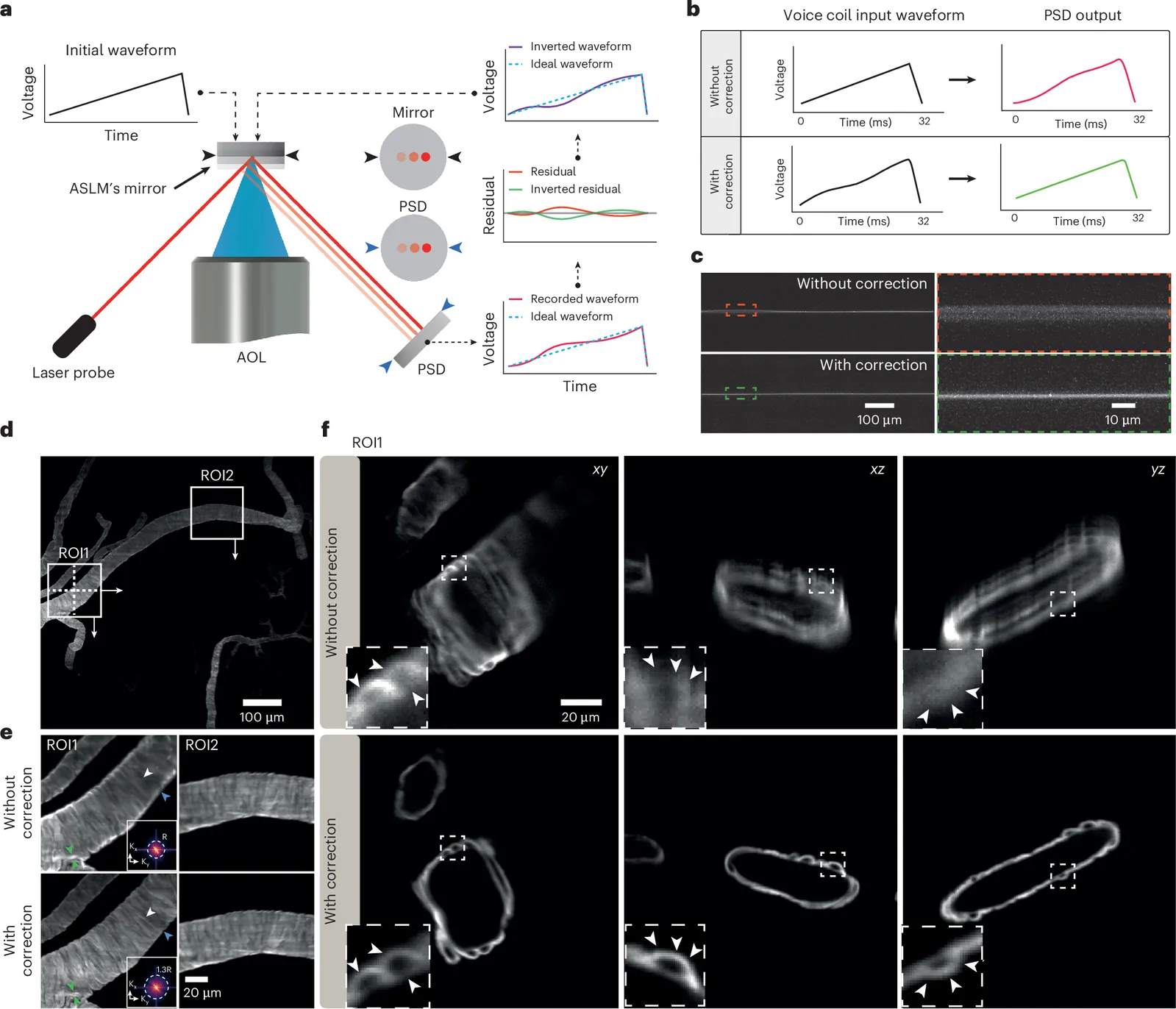
High-speed isotropic resolution achieved by voice coil waveform adaptation. **a**, Schematic of the PSD arrangement and the closed-loop control of the voice coil in the ASLM unit, along with the adaptive algorithm for correcting voice coil movement. PSD indicates the position-sensing device. **b**, Recorded feedback waveform from the PSD without and with correction at voice coil frequency 31 Hz. **c**, Recorded swept beam inside the chamber corresponding to the waveforms in **b** with an exposure time of 25 ms. **d**, Recorded mouse brain stained for αSMA, showing a selected ROI with and without waveform correction. **e**, Selected ROI1 and ROI2 in **d**. Resolution analysis in the Fourier domain shows an increase in resolution after voice coil correction. **f**, Cross-section of ROI1 from **d** in the *xy*, *xz* and *yz* planes with and without voice coil movement correction. The subsets and arrowheads highlight the same area. The images in **d**–**f** are examples from one of four sampled images.

Alongside spatial resolution, imaging speed is also important for achieving high-throughput imaging of large samples. In ASLM, the maximum speed is determined mainly by the sweeping device, which in our case is a voice coil with a reported speed of up to only 10 Hz for ASLM^13^. To explore the voice coil’s potential to move much faster, we developed a small, closed-loop monitoring arm with a low-power diode laser and a position-sensing detector (PSD; Fig. 3a). The axial movement of the voice coil translated into a lateral shift of the reflected laser on the PSD and a modulation of its voltage output (Methods). Our measurements across typical exposure times from 8 ms to 256 ms confirmed that the voice coil’s response was not uniformly linear across all frequencies, particularly at higher frequencies, which desynchronized the movement of the light sheet and the rolling shutter at several parts of the FOV (Supplementary Fig. 5). To eliminate the nonuniformity, a correction waveform was calculated based on the residual of the voice coil’s response to the initial sawtooth waveform, which was then applied back on the voice coil as a correction waveform. Notably, even after just a few iteration cycles, the voice coil’s movement became uniformly linear, allowing for precise synchronization between the sweeping light sheet and the sCMOS rolling shutter at almost full speed without compromising FOV coverage (Fig. 3b,c). This approach also allowed us to synchronize the voice coil with the rolling shutter at a frame rate of 100 frames per second (fps) (Supplementary Table 1).

We next tested the system’s performance for high spatiotemporal resolution imaging of a piece of cleared mouse brain (800 μm × 800 μm × 2,400 μm) with fluorescently labeled larger vessels, especially arteries and arterioles, using an antibody detecting smooth muscle cells (α-smooth muscle actin, αSMA; Fig. 3d). Here, the imaging was done with an sCMOS camera with a controllable rolling shutter at its maximum frame rate (PCO Panda; Supplementary Table 1). The recorded data showed a resolution variation across the FOV and along the sweeping light sheet in both lateral and axial views before the voice coil waveform correction. Notably, the isotropic resolution across the entire FOV was achieved only with the corrected waveform of the voice coil. Individual fine vasculature structures were resolved in all directions at submicron scale at 40 Hz across the entire FOV (Fig. 3e,f).

### Benchmarking the light-sheet system by imaging cleared specimens at different RIs

**Fig. 4:**
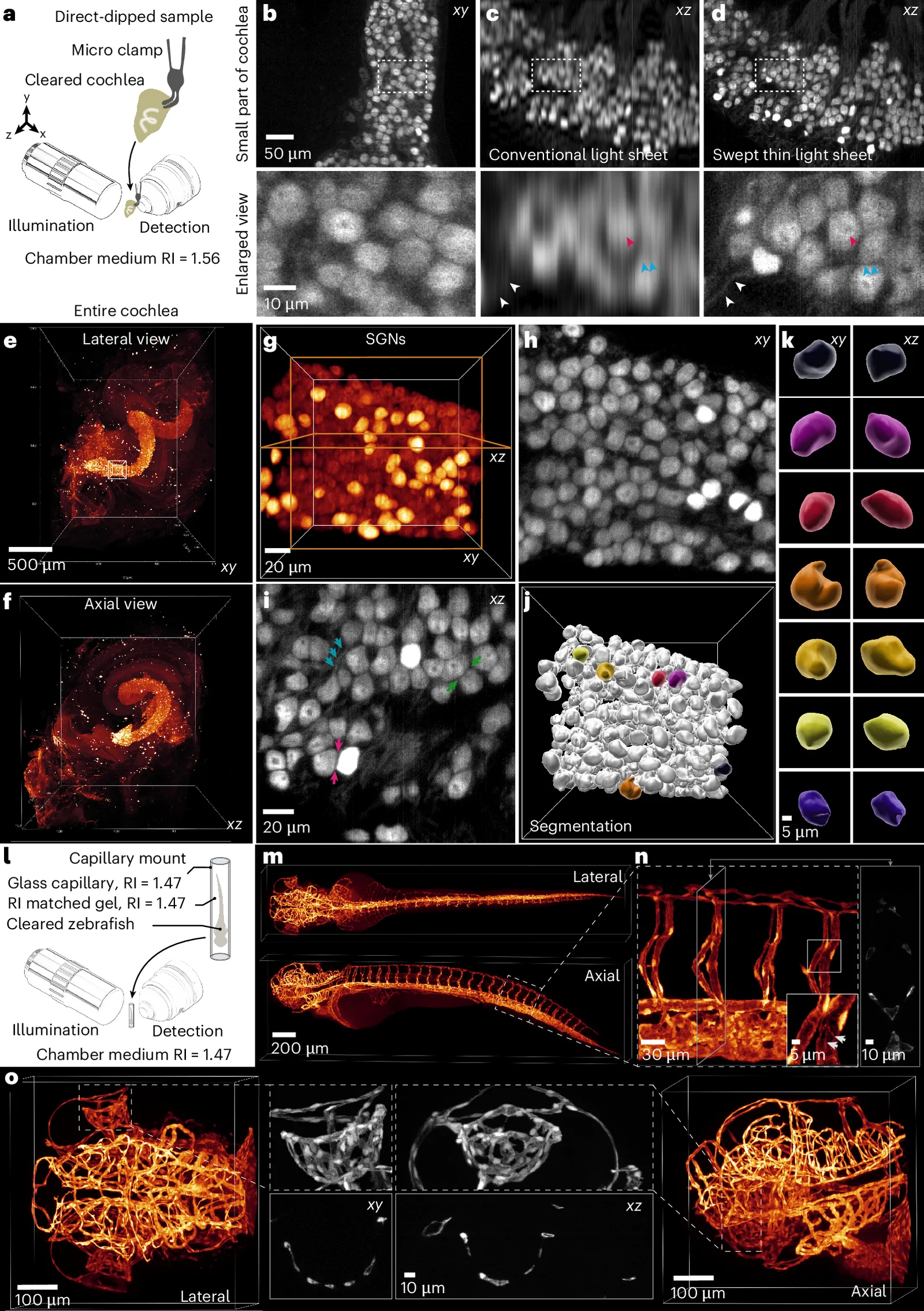
Imaging of a mouse cochlea with isotropic resolution. **a**, Schematic of the cochlea and its mounting in the microscope. **b**, Top: recorded data from a small section of an anti-PV-stained cochlea with a swept thin light sheet. Bottom: selected and magnified ROI from the top row. The image is an example from one of two imaged samples. **c**, Top: reconstructed axial image in **b** recorded with a conventional light sheet covering the entire FOV. Bottom: selected and magnified ROI from the top row. **d**, Top: reconstructed axial image in **b** recorded with a swept thin light sheet. Bottom: selected and magnified ROI from the top row. All arrows of the same color indicate comparisons between the magnified images in **c** and **d**. **e**, 3D reconstruction of a mouse cochlea from four tiles (lateral view). The image is an example from one of five imaged samples. **f**, 3D reconstruction of **e** (axial view). **g**, An ROI in the cochlea base with SGNs from **e**. **h**,**i**, Lateral (**h**) and axial (**i**) view of the midplane in **g**. Blue arrows mark exemplary neurites of the bipolar SGNs. The green and red arrowheads indicate the well-resolved gap between the neurons due to the isotropic resolution. **j**, 3D rendering after segmentation in **g**. **k**, Selected segmented SGNs in **j** (lateral and axial view), labeled with a unique color for each SGN. **l**, Schematic of the zebrafish and its mounting in the microscope using a borosilicate glass capillary. **m**, 3D reconstruction of the zebrafish from both lateral and axial views. The image is an example from one of four imaged samples. **n**, Enlarged view of a selected ROI in the tail; the inset highlights magnified vessels within this ROI. All arrows indicate the endothelial cell boundaries. The right panel displays a cross-section of the tail. **o**, Enlarged views of the zebrafish head from **m**, including lateral and axial views (first and last). The middle images represent maximum intensity projections of the vasculature around the eye in both orientations, along with a central cross-section.

To benchmark the microscope’s performance for rapid and isotropic imaging of whole cleared organ samples, we first imaged an entire mouse cochlea. The cochlea offers an intricate snail-shaped three-dimensional (3D) structure with the spiral ganglion sampling sound information along the frequency axis. iDisco cleared mouse cochlea^10^ (Methods) were matched with an RI of 1.56 (ECi; Fig. 4a). Immunolabeling with parvalbumin (PV) allows for robust visualization of the entire population of spiral ganglion neurons (SGNs) within the Rosenthal canal. We first imaged a small part of the cochlea to see how much more information can be retrieved along the axial direction when using a thin swept light sheet compared to a conventional light sheet with a thickness of about 5 μm covering the entire FOV: detailed observation, especially in the axial direction, had hitherto remained elusive when using conventional light-sheet methods (Fig. 4b–d). The typical volume of a cochlea is approximately 1.5 mm × 1.5 mm × 2 mm (Fig. 4e,f) and could be imaged in four tiles. We employed a plane spacing of 380 nm, consistent with the pixel size, for an RI of 1.56 to achieve a resolution of 850 nm. Up to 4,000 imaging planes were needed in each tile to cover the entire volume. Because of the isotropic resolution, each SGN was clearly distinguished from its neighbors, and neurites were detected (Fig. 4g–i), enabling us to segment each SGN properly (Fig. 4j,k). Alongside submicron isotropic resolution, our microscope allowed us to image a mouse cochlea with four tiles in just 2.1 to 8.5 min at an exposure time of just 8–32 ms, respectively (Supplementary Video 3).

To evaluate the microscope’s performance at different RIs, we cleared a 3 days postfertilization (dpf) Tg(kdrl:GFP) zebrafish using an adjusted version of the EZ Clear protocol^20^ (Methods). The zebrafish was mounted in an RI-matched agarose gel (RI = 1.47) inside a borosilicate glass capillary suspended in glycerol (Fig. 4l). This mounting method allows for easy mounting of delicate or fragile samples while maintaining a uniform RI (Methods). The sample was imaged in four tiles, totaling 8,000 planes for the entire sample, with a plane spacing of 390 nm. The entire sample is recorded with 100 fps within an acquisition time of 1 min and 20 s (Fig. 4m–o). The data showed consistent image quality in both lateral and axial views, with fine details such as endothelial cell boundaries clearly resolved in all directions (inset; Fig. 4n). Isotropic resolution was confirmed by examining the delicate ophthalmic vasculature in both lateral and axial views, which showed equally well-resolved vascular walls and lumina (Fig. 4o and Supplementary Video 4).

### Isotropic 3D imaging enables analysis of cochlear connectomics

**Fig. 5:**
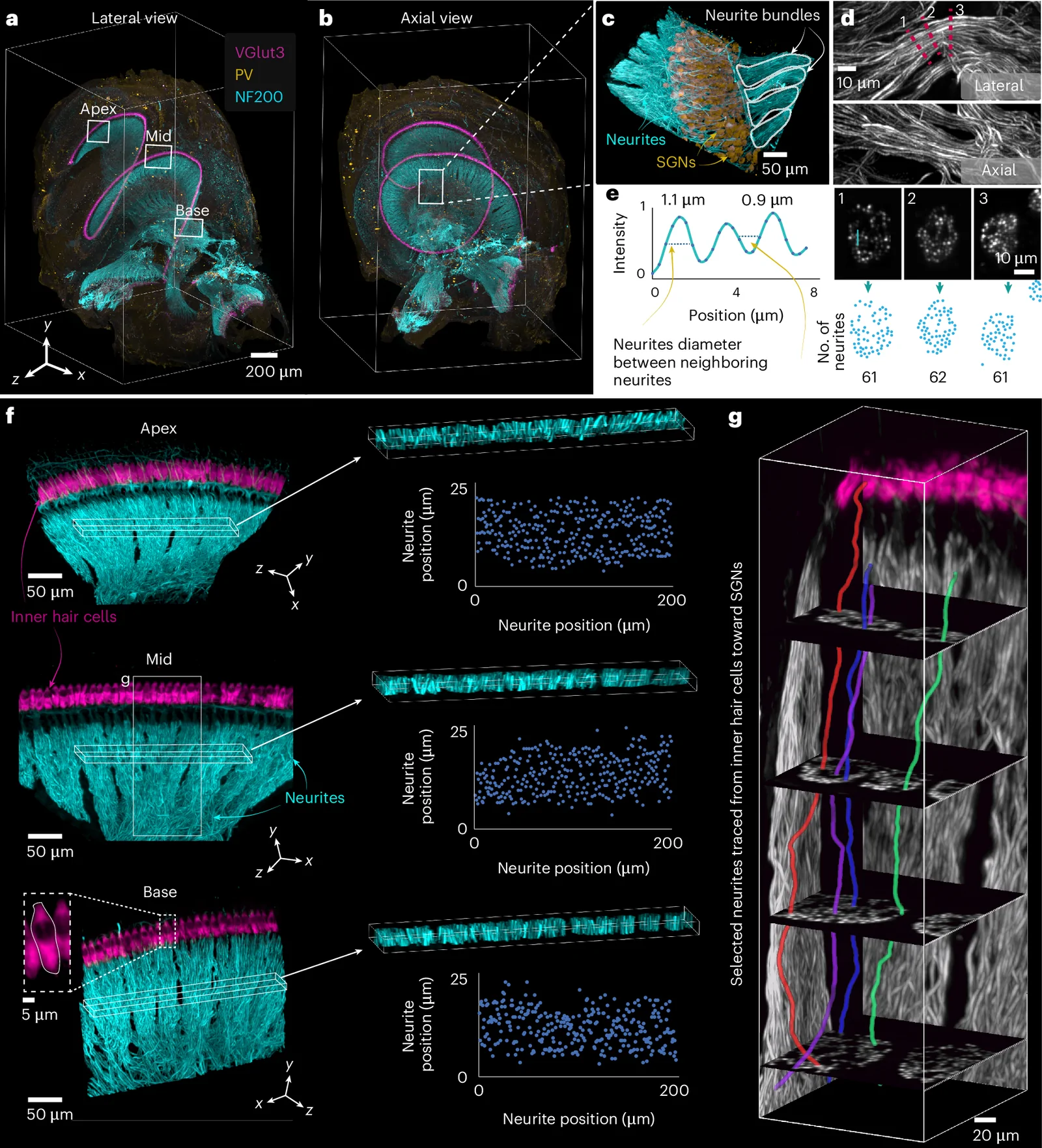
Multicolor imaging of the mouse cochlea. **a**,**b**, Lateral (**a**) and axial (**b**) views of a full cochlea volume showing IHCs immunolabeled for VGlut3, SGNs labeled with anti-PV and neurites labeled with NF200 in *x*, *y* and *z*. The image is an example from one of three imaged samples. **c**, Selected and magnified ROI from **b**, showing neurites and SGNs (left) and neurites in lateral and axial views (right). **d**, Analysis of a single neurite bundle in **c**. First row: lateral view; second row: axial view; third row: neurite diameter and position in three oblique planes along the neurite extension marked with a red dashed line and numbered 1, 2, 3; fourth row: neurite count from exemplary cross-sections shown in **d**, first row. **e**, Line profile of three neighboring neurites in cross-section 3 in **d**. **f**, Selected ROIs from the apical, middle and basal turns of the cochlea, as shown in **a**, depicting IHCs labeled with VGlut3 and neurites labeled with NF200 to visualize neurite positions for exemplary counting across all cochlear turns. **g**, Tracing SGN neurites from the organ of Corti to the spiral ganglion in an ROI from the midturn of the cochlea, shown in **d**.

To demonstrate the biological applicability and resolution performance of our light-sheet microscope in structured and functionally critical tissue, we performed high-resolution imaging of cochlear afferent innervation. Using triple-color fluorescent labeling, we visualized inner hair cells (IHCs), SGNs and their peripheral neurites (Fig. 5a,b). Imaging was carried out at the system’s maximum isotropic resolution, enabling a comprehensive 3D reconstruction of cochlear architecture with consistent detail across all spatial orientations. We first rendered whole-organ 3D overviews with clearly separated channels, followed by the selection of a region of interest (ROI) from the basal turn encompassing SGNs and their neurites (Fig. 5c–e and Supplementary Video 5). Within this ROI, we extracted three cross-sectional planes perpendicular to the local neurite trajectory at distinct locations along the cochlear spiral. In these planes, individual neurites could be resolved and counted, indicating that fine anatomical structures remained sharply defined regardless of orientation. This underscores the system’s isotropic resolution and its suitability for neuroanatomical quantification.

To expand the analysis, we selected three larger ROIs from the apical, middle and basal turns. In each, we quantified the number of SGN neurites terminating on 25 adjacent IHCs—a direct proxy for IHC–SGN connectivity, which is critical for understanding auditory function and pathology (Fig. 5f). The ability to perform these counts across the cochlea consistently reflects the uniform image quality, enabling comparative assessments of innervation density and spatial patterning along the tonotopic axis.

We then traced and segmented selected neurites from their synaptic connection with IHCs to the corresponding SGN soma within the Rosenthal’s canal, capturing their full 3D trajectories (Fig. 5g). This level of structural detail supports studies of cochlear neuronal connectivity, allowing reconstruction and analysis of afferent pathways at the single-cell level. Such detailed mapping opens the door to systematic studies of cochlear connectomics, including afferent synaptic organization of type I and II SGNs, molecularly defined type I SGN subtypes^29–31^, efferent connectivity, and circuit-level remodeling in health and disease.

### Imaging of the whole mouse brain

**Fig. 6:**
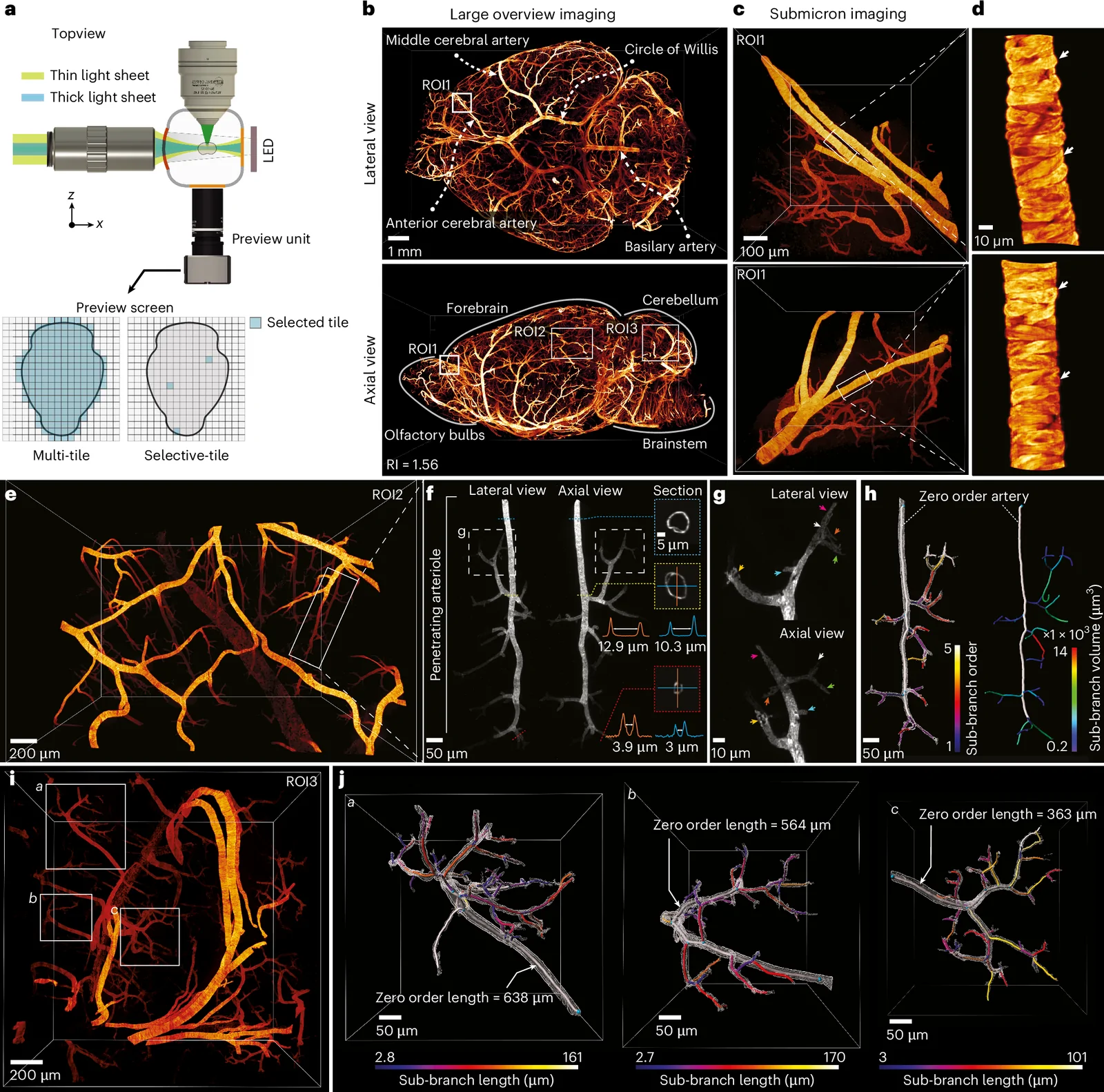
Hybrid imaging of an entire cleared mouse brain. **a**, Schematic of the setup with chamber, lenses and a small camera unit for capturing a preview image of a large sample and selecting the tiles. **b**, Entire reconstructed mouse brain from lateral and axial views. The image is an example from one of two imaged samples. **c**, Re-imaged selected ROI in **b** and the reconstructed axial view (second row). **d**, Enlarged view of selected small vessel in **c** (first row) and reconstructed axial view (second row), highlighting individual vessel cells (arrowheads). **e**, Selected ROI2 from the cortex in **b**, which was recorded with the highest possible isotropic resolution. The image is an example from one of two imaged samples. **f**, Selected penetrating arterier and cross-sections at different depths and branches. The vertical line (orange solid line) and horizontal line (blue solid line) profiles indicate the measured line profiles from the cross-sections of the zero-order artery and the last SMA-positive termini. The inner distance between the artery walls was measured and indicated with a white double arrowhead line. **g**, Enlarged view of one of the penetrating subarteries in **f**. The color-coded arrowheads indicate the last SMA-positive arterial termini with the same color from both lateral and axial views. **h**, Left: segmentation of the entire penetrating artery and the counted sub-branches until the last SMA-positive termini. Right, the calculated volume of the main and subarteries. **i**, Selected ROI3 from the cerebellum in **b**, which was recorded with the highest possible isotropic resolution. The image is an example from one of two imaged samples. **j**, Full segmentation of three selected penetrating arteries named *a*, *b* and *c*. The segmentation and artery tracing enabled us to find the length of the main and subarteries in all directions.

To assess the system’s performance in imaging even larger specimens, we imaged an entire mouse brain (14 mm × 10 mm × 7 mm) immunolabeled for arteries and arterioles by αSMA-targeting antibodies (Supplementary Fig. 6; Methods). A compact preview system with a ×0.3 optical magnification was installed opposite the detection lens, providing a large overview of approximately 15 mm × 15 mm (Fig. 6a). The preview assists the user in choosing the desired orientation and selecting several tiles across the entire sample, each covering a full detection FOV of 800 μm × 800 μm.

We first acquired a quick overview of the mouse brain by selecting all the tiles covering the brain and imaging them with a thick light sheet (3.8 μm). This reduction in axial resolution is achieved quickly by limiting the NA of the illumination beam with an adjustable slit. To achieve an isotropic overview, the pixel size of the recorded image was then matched to the light-sheet thickness by a binning of ten. The entire mouse brain was recorded and reconstructed at this resolution in less than 30 min. The reconstructed data enabled us to identify the entire brain’s vasculature trees, including the main arteries located at the surface as well as deeper arterioles (Fig. 6b). To observe the vessels’ subcellular structure, we identified ROIs, for example, part of the anterior cerebral artery, and re-imaged the related selected tile volume (800 μm × 800 μm × 800 μm) again at the highest possible isotropic resolution (850 nm), which only took about 21 s for 2,100 layers (Fig. 6c). The raw data provided isotropic subcellular information of individual smooth muscle cells viewed from any angle without deconvolution or other processing (Fig. 6d and Supplementary Video 6).

To further benchmark the biological relevance and resolution fidelity of the system, we imaged two distinct parts of the mouse brain, targeting anatomically and functionally different regions: the cerebral cortex and the cerebellum. Imaging was carried out at the maximum achievable resolution, allowing us to visualize fine vascular structures throughout the entire volume without compromising depth or FOV. In each region, we isolated and analyzed three individual penetrating arteries, each differing in depth and spatial orientation. We were able to accurately measure vessel diameter along their full trajectories, regardless of spatial orientation or local deformation—demonstrating the system’s ability to maintain uniform, isotropic resolution. This overcomes a main limitation of conventional LSFM systems, which often suffer from degraded axial resolution (Fig. 6e,f).

Using 3D segmentation and skeletonization, we reconstructed the full branching architecture of each vessel down to the last SMA-positive termini (Fig. 6g). Precise illustration of the arteriolar-capillary transition zone allowed for clear distinction of different types of mural cells without additional markers or tissue cutting. The ring-like morphology of smooth muscle cells in the arterioles was replaced by an enwrapping morphology in higher-order vessels, reflecting ensheathing pericytes^32^. Notably, mural cells in higher-order branches appeared less intense, suggesting a reduced expression of αSMA in those cells. The full reconstruction enabled precise quantification of branch number, length and morphology (Fig. 6h and Supplementary Fig. 7), supporting applications that rely on high-fidelity structural analysis, such as vascular remodeling, angiogenesis during development or vascular remodeling after ischemic insults.

In the cerebellum, vascular features—including the lengths of penetrating arterioles, branching patterns and compact geometries—were also readily recorded. These characteristics, which differ from those in the cortex and reflect the anatomical and functional distinctions of this brain region, were visualized without the need for angle-specific imaging, highlighting the versatility and consistency of our system (Fig. 6i,j).

Overall, this analysis highlights how our microscope enables orientation-independent, subcellular-resolution imaging across large tissue volumes. The ability to accurately trace and quantify individual vessels in three dimensions, regardless of geometry or orientation, makes it a powerful tool for investigating brain vasculature, microcirculation and disease-associated structural changes at unprecedented clarity and scale.

## Discussion

High-resolution 3D imaging and quantitative analysis of large biological samples are essential for understanding complex structures such as neuronal circuits and vascular networks. Maintaining spatial context throughout the entire volumes allows for precise and efficient extraction of morphological and functional insights. To meet these needs, we have developed a rapid, aberration-corrected light-sheet microscope for imaging cleared tissues with isotropic submicron resolution. This approach demonstrates that detailed morphometric and connectivity analysis across whole specimens has now become possible without requiring tissue reorientation, flattening, distortion or sectioning.

The microscope uses an axially swept light sheet to achieve isotropic resolution over a large FOV. We found that the light-sheet field curvature can be effectively corrected by replacing the flat mirror in the ASLM with an off-the-shelf concave mirror rather than using an expensive, dedicated objective lens. The straightened light sheet is key to achieving an isotropic PSF across the extended FOV and stitching volume tiles accurately. Although we have corrected the field curvature only in our illumination arm, the same principle could be extended to remote focusing in the detection arm for fast volumetric imaging, as sometimes required in live imaging.

Furthermore, we minimized spherical aberrations and achieved a near-diffraction-limited light sheet using identical, easily exchangeable high NA air objective lenses in both the illumination and ASLM arm. Combined with an off-the-shelf meniscus lens, we achieved submicron resolution. Aberrations in the illumination arm are minimized because both objective lenses are identical. Again, this concept represents a cost-effective solution that could also be extended to the detection arm in future developments. The curvature of the meniscus lens is chosen to match our primary NA of 0.4, but can be adapted to other lenses as needed.

Our closed-loop technique for calibrating and monitoring the ASLM unit enables us to achieve the very high frame rate of 100 Hz. The system controls several aspects of operation, such as precalibrating the voice coil’s running waveform and monitoring the voice coil’s movement for any drift, for example, due to environmental factors such as temperature. Our technique can adapt to various voice coil frequencies, such as the typical sCMOS with 40 Hz, 100 Hz and 122 Hz frame rates in light-sheet mode (Supplementary Table 1), making it universally applicable and robust. Further advancements in sCMOS technology with additional light-sheet modes and potentially higher frame rates can be incorporated without sacrificing FOV. Furthermore, our approach may apply to other microscopy techniques that rely on precise remote focusing^33^.

The striping artifacts typically associated with light-sheet microscopy are less pronounced when samples are well-cleared and RI-matched as shown. However, residual striping artifacts can be mitigated using multidirectional selective plane illumination microscopy (mSPIM)^34^ or computational methods^35^. The mSPIM could be integrated seamlessly into our microscope by employing a fast resonance mirror or an acousto-optic deflector (AOD) with kilohertz-level scanning. Nevertheless, some samples may not achieve perfect RI matching due to hard tissues like bone^36^, which may shift the light sheet and detection plane from the perfect focus^37^. We have addressed this issue by axially fine-tuning the light-sheet position through voice coil offset adjustments and defocusing the light sheet to the detection plane using a motorized mirror placed after the CL. Finding the optimal imaging angle may become important in future developments, potentially leveraging smart rotation^38^. Although our proposed method has been applied within a conventional light-sheet configuration with two objective lenses, all techniques introduced in this study can be adapted readily to various other light-sheet configurations with different numbers of lenses for diverse applications, including live tissue imaging.

In summary, our cleared tissue microscope is a robust, compact system built from commercially available, off-the-shelf components. We achieved isotropic, submicron resolution in all directions with high-speed imaging for high-throughput imaging of biological samples. Our light-sheet microscope can reveal the complex subcellular architecture across large intact tissues, providing new insights for biological and medical research.

## Methods

### Optical setup

The optical setup consists of six units: laser launch, remote focusing, illumination arm, chamber, detection arm and preview unit.

1. Laser launch: the setup can either be used with a fiber-beam laser engine (CLE laser engine, 20 mW each laser line, TOPTICA) or a free-beam laser engine (OBISCellX, 100 mW each laser line, Coherent). Each laser engine provides four laser lines at 405 nm, 488 nm, 561 nm and 640 nm. When using a fiber-coupled laser, the laser beam is collimated with a 25-mm lens, which gives a 7-mm beam. For the free laser beam, a lens with *f* = 50 mm lens (where *f* denotes the focal length) is paired with a lens with *f* = 25 mm to provide a twofold beam expansion, which also provides a beam with a 7-mm diameter at the 561-nm laser line. Following the beam collimation and expansion, an initial light sheet is formed with a CL with *f* = 45 mm. This primary light sheet is subsequently collected by a secondary lens with *f* = 60 mm. A polarizing beam splitter (PBS) that guides the beam through the ASLM arm is positioned in front of this secondary lens.
2. Remote focusing: the beam polarization is controlled by rotating the optical fiber to achieve maximum reflection at the PBS toward the ASLM. After passing the PBS, the laser beam encounters a sequence of optical elements: a quarter-wave plate (QWP), a 100-mm lens, a mirror, and a ×20 objective lens before hitting the ASLM mirror. The QWP converts the laser beam’s linear polarization into circular polarization (for example, clockwise). After reflection from the ASLM mirror, the beam returns with a circular polarization in the opposite direction (counterclockwise). When the beam passes through the QWP again, it regains linear polarization but in an orthogonal direction, enabling it to pass through the PBS and proceed to the IL. The ASLM unit has a voice coil actuator (Thorlabs Blink) to sweep the light sheet. This setup includes either a flat mirror with a diameter of 12.5 mm (Thorlabs) or a concave mirror (Thorlabs, cat. no. CM127-010-P01) with a 9.5-mm focal length, which corrects the light-sheet field curvature while simultaneously sweeping the light sheet.
3. Illumination arm: following the PBS toward the main illumination arm, a 60-mm focal length lens collects the reflected beam after the remote focusing. The lens back focal plane is conjugated to the PBS center, while its front focal plane aligns with the IL back focal plane (Mitutoyo ×20 NA 0.4, cat. no. MY20X-804). After the illumination lens, the beam encounters a positive meniscus lens with a 100-mm focal length (Thorlabs, cat. no. LE1234). This lens acts as a correction plate, preventing spherical aberrations when transitioning from air to a medium with a higher RI in the chamber.
4. Chamber: the chamber is made out of aluminum with four openings. One is used for holding the meniscus lens (Thorlabs, cat. no. LE1234). Two of them hold two glass windows with a thickness of 1 mm (Thorlabs, cat. no. WG10110), one for the preview camera and one for the preview LED. The last opening is used for the dipping detection objective lens. All the glass and lenses are sealed with sealant O-rings.
5. Detection unit: the detection unit uses a multi-immersion dipping objective lens (ASI multi-immersion lens ×16 NA 0.4, 15-10-12) with a working distance of 12 mm at RI 1.45. The emission light is filtered through one of the six emission filters mounted on a custom-made filter wheel equipped with a robotic servomotor. For standard operation, the image is formed by a 200 mm TL (Thorlabs, cat. no. TTL200-A) onto an sCMOS chip with a rolling shutter (PCO Panda 4.2m or Hamamatsu Fusion, cat. no. C14440-20UP) featuring a 6.5-μm pixel size and a 2,048 × 2,048 pixel square chip. Alternatively, a 160-mm tube lens was used for an sCMOS chip with a 4.6-μm pixel size (PCO 10bi CLHS).
6. Stage and sample holder: for volumetric imaging, the specimen is moved continuously through the light sheet in the *z* direction. This process captures a block tile using a linear stage (PI M112) at plane spacings of 0.38 μm, mirroring the detection system pixel size, when the RI is at 1.56. Here we used tiles in the *x* and *y* dimensions for specimens that exceed the detection system’s FOV, using another two linear stages (PI M112).

### RI compatibility

The meniscus lens (positive meniscus, focal length 100 mm) was selected to match the curvature of the illumination beam’s wavefront, which is determined by the NA of the illumination objective. This design ensured that the beam enters the sample chamber from air into higher RI media at a nearly perpendicular angle across the entire beam profile. Consequently, the performance of the meniscus lens remained unaffected by the RI of the chamber medium, making it compatible with all common clearing protocols. Similarly, the concave mirror used in the ASLM path compensated for the field curvature introduced by the overall optical setup of the illumination arm. Its function is also independent of the chamber RI. Therefore, switching between different immersion media requires no replacement of the meniscus lens or the concave mirror.

### Compensation of chromatic aberrations for multiwavelength imaging

We used an air objective lens in the illumination arm, which, in combination with the high RI of the clearing solvent, can introduce chromatic aberrations. This resulted in wavelength-dependent shifts in the position of the light sheet along the sweeping direction. To correct for this shift and ensure that all wavelengths focus at the same position, we experimentally determined the optimal offset voltage for the voice coil actuator at each excitation wavelength. As we performed multicolor imaging sequentially, we loaded a separate, precalibrated waveform for each laser wavelength, each with the appropriate offset voltage applied. This ensured that light sheets of different wavelengths are aligned spatially with the rolling shutter, enabling accurate multicolor imaging without chromatic aberration.

### Data streaming, postprocessing and visualization

The microscope is operated by a compact computer (NUC 11) running on an Ubuntu operating system. The data collected by the sCMOS is streamed seamlessly from the NUC to an external solid-state drive (Samsung T7, 4TB) via a Thunderbolt connection during standard operations, maintaining a frame rate of under 40 fps. For higher frame rates exceeding 40 fps, a 2TB M2 (Samsung NVMe) drive housed in an enclosure that supports Thunderbolt 4 is used, allowing for a streaming speed of 40 Gb per second. Each imaged volume tile is stacked directly in BigTiff or RAW format as it is written to the external solid-state drive. Subsequently, the data is converted into HDF5 format to enable lossless compression for Fiji^39^ (v.1.54p) and BigStitcher^40^ (v.2.5.2) for further analysis and image stitching. All 3D rendering and segmentation have been done using Imaris (v.9.7).

### PSF measurement

The microscope’s PSF was measured by imaging 200-nm gold nanoparticles (Aldrich, cat. no. 742066, lot no. MKCQ8675) embedded in clear, RI-matched agarose gel. First, 1.5% hard-gelling agarose is prepared with 0.2% gold nanoparticles already vortexed for about 5 min to avoid clustering. It is mixed with the prepared agarose gel and formed with a 2 mm glass capillary in a cylinder shape for easy holding. Once solidified, the gel is pushed out with a plunger into ethanol for dehydration. The dehydration process is done in three steps using 20%, 50% and 100% ethanol for 15 min, 15 min and 30 min, respectively. After dehydration, the agarose gel is transferred to the final RI-matching solvent for at least 1 day. Once RI-matched, the agarose is transferred to the same capillary or glued to a sample holder for PSF measurement. For the PSF measurement, we imaged the nanoparticles using a 561-nm laser line with a power of 0.5 mW, a *z*-step size of 380 nm, and a 20-pixel camera rolling shutter. All parameters corresponded to the same slit size and *z*-step as used for imaging the biological samples. The PSF is then determined based on the full-width at half-maximum of the intensity profiles of the recorded nanoparticles using Fiji^39^.

### Closed-loop monitoring arm with a PSD

We have used a PSD (Thorlabs, cat no. PDP90A) with a K-Cube controller (Thorlabs, cat. no. KPA101) in combination with a compact probe laser module with a Gaussian intensity profile (1 mW laser, 635 nm; Thorlabs, cat. no. PL202). The PSD and probe laser are positioned at 45 degrees relative to the optical axis of the ASLM unit. The laser is reflected on the ASLM mirror and hits the PSD. The axial movement (along the optical axis) of the voice coil translates into the lateral movement of the laser spot on the PSD. By tracking the center position of the laser beam on the PSD, the output voltage of the K-Cube controller (Output X) generates a corresponding signal with an accuracy of 0.7 μm at a wavelength of 635 nm (approximately 100 μW at the PSD). To achieve this level of accuracy, we have used an absorptive neutral density filter with 10% transmission (Thorlabs, cat. no. NE510A, half-inch diameter) attached to the PSD. It should be noted that the closed-loop monitoring is used only during calibration to optimize the voice coil waveform for the desired scan frequency. During actual imaging, the system operates with the precalibrated waveform.

The output of the K-Cube is connected to a compact multi-function generator (Moku:Go, Liquid Instruments), which streams the PSD output directly to the controlling computer for further analysis (Supplementary Fig. 8). The signal is then processed using a Python script that calculates the residual of the PSD output compared to the initial linear waveform and generates a corrective waveform. The corrective signal is sent to the voice coil via a function generator. The system operates using a feedback closed-loop algorithm, which works by either converging to a predefined metric or incorporating user feedback based on the swept beam quality (Supplementary Note 4). The best corrective waveform is selected, typically achieved in fewer than ten iterations. Ultimately, the optimal corrective waveform for each target frequency needs to be calculated only once.

### Field curvature measurement

To measure the light-sheet field curvature, we used the PSF measurement and direct imaging of the light-sheet cross-section. The PSF measurement was the same as described. However, for the light-sheet cross-section measurement, we used a small mirror of 2 mm × 2 mm glued to a glass capillary. The mirror is placed in the chamber between the objective lenses at 45 degrees, directly reflecting the light sheet into the detection arm. Without any field curvature, the cross-section of the light sheet should be a straight line from top to bottom of the FOV. However, in the presence of field curvature, the light-sheet thickness varies along the FOV height. To measure the field curvature, we first parked the light sheet in the center of the detection FOV (the voice coil was parked at the center position with zero amplitude). Then, the light-sheet cross-section is recorded in several positions by gently moving the mirror along the FOV. The intensity profile of the light sheet for recorded cross-sections is measured from top to bottom. Then, the thinnest light sheet in each plane along the FOV height is selected. The relation between these thinnest parts of the light sheet describes the field curvature.

### Animals

Animal handling complied with relevant national and international guidelines for the care and use of laboratory animals in research (European Guideline for Animal Experiments 2010/63/EU) and in accordance with regulations put forward by the state authorities of Lower Saxony (LAVES) and Schleswig-Holstein (MLLEV). The mice were kept at a constant temperature (22 °C) and humidity (50%) on a 12-h light/dark cycle and were provided with standard laboratory chow and water ad libitum as well as enrichment. Mice euthanized for extracting cochleae and brains in this study aided the development of the new microscope presented here, capable of high-throughput and high-resolution submicron imaging to further address various biological questions in following studies. Mice for brain extraction were bred at the local animal facility of the University of Lübeck. Male C57BL6/N mice at the age of 4–6 months were used. Adult zebrafish (*Danio rerio*), embryos and larvae were maintained following established protocols. Adult fish were kept at a water temperature of 26.0 °C and a 14 h/10 h light/dark cycle. Fish were fed standard laboratory food supplemented with live *Artemia* larvae. The transgenic line *Tg(kdrl:GFP)* was used, where green fluorescent protein (GFP) is expressed in the vascular endothelium. Zebrafish embryos were kept at 28.0 °C in 0.3× Danieau’s medium with 0.003% 1-phenyl-2-thiourea added at 1 dpf to suppress pigmentation. Embryos were checked for potential malformations and expression of the endogenous reporter using a fluorescence stereomicroscope. Larval zebrafish at 3 dpf showing the desired expression pattern and without apparent malformations were selected for sample preparation.

### Sample preparation (mouse cochlea)

After extraction from the temporal bone, cochleae were fixed for 45 min in 4% formaldehyde, subsequently rinsed in phosphate-buffered saline (PBS) and decalcified for up to 1 week in 10% ethylenediaminetetraacetic acid (pH 8). During decalcification, cochleae were trimmed continuously with surgical instruments (Fine Science Tools) using a bright field microscope (Zeiss; Olympus SZ61) to remove as much bone as possible for easy access and later penetration of the light-sheet beam during optical sectioning.

### Immunolabeling and tissue clearing (mouse cochlea)

For robust and reproducible immunolabeling as well as optical clearing, we used a newly adapted version of the optimized iDISCO^+^ clearing protocol adapted to the cochlea^10^ (cDisco^+^). Here we first decalcified the mouse cochlea for 4 days using 10% ethylenediaminetetraacetic acid of pH 8.0 at room temperature. Second, we decolorized the decalcified specimen for up to 3 days at 37 °C using 25% amino alcohol (N,N,Nʹ,Nʹ-Tetrakis(2-Hydroxypropyl)ethylenediamine). Next we employed pretreatment and permeabilization of the samples for approximately 4 days according to iDisco^19^, specifically without the use of methanol, followed by epitope blocking at 37 °C. For reliable immunolabeling of the entire population of SGNs within the Rosenthal canal, sufficient penetration of the cochlea was achieved by incubating each antibody for 14 days at 37 °C followed by washing in PBS-Tween 0.2% with Heparin 10 μg ml^−1^ for up to 1 day^10^. Here we used a guinea pig polyclonal anti-PV antibody (Synaptic Systems, cat. no. 195 004; lot no. 3-38; clone not specified) at a dilution of 1:300, preconjugated with a camelid single domain anti-guinea pig antibody labeled with Atto565 (NanoTag Biotechnologies, cat. no. N0602; lot no. 022205; clone not specified) at a molar ratio of 1:3. Further, we used a monoclonal mouse anti-neurofilament 200 (NF200) antibody (Sigma-Aldrich, cat. no. N5389; lot no. 099M4843V; clone NE14) at a dilution of 1:400 and a polyclonal rabbit anti-vesicular glutamate transporter 3 (VGlut3) antibody (Synaptic Systems, cat. no. 135 203; lot no. 1-47; clone not specified) at a dilution of 1:300. For a secondary staining staining an anti-mouse antibody labeled with Alexa 647 (Invitrogen, cat. no. A-21236; lot no. 751096; clone not specified) and an anti-rabbit antibody labeled with Alexa 488 (Invitrogen, cat. no. A-11008; lot no. 659082; clone not specified), each at a dilution of 1:200 were used. Following the immunolabeling, the cochleae were dehydrated, stepwise, in a methanol/H_2_O series ranging from 20% to 100% methanol for up to 1 day and then delipidized with dichloromethane at room temperature^41^. Finally, RI-matching was performed by initial embedding in dibenzyl ether for 1 day and consecutive transfer to ECi for storage and imaging at room temperature.

### Sample mounting (mouse cochlea)

For imaging and sufficient matching of the RI the cleared cochleae remained in ECi for at least 6 days. The cochleae were then mounted carefully on a customized 3D-printed sample holder. Here we clamped the cochlea dorsal of its basal turn at the attached vestibular system to the holder and inserted the mounted cochleae with the cochlear apex facing the bottom of the imaging chamber. Thus, the modiolus of the cochlea is placed approximately perpendicular to the applied light-sheet beam within the imaging chamber.

### Mouse brain

The whole procedure of preparing, staining and clearing the tissue was performed according to a published protocol^42^ with slight adaptations. The method is based on the iDISCO procedure and optimized to visualize the brain vasculature. Briefly, after decapitation, the samples were extracted by opening the skull and removing the brain, ideally including the meninges and surface vessels. In addition to perfusion fixation using 4% paraformaldehyde in PBS, a postfixation period of 3 h at room temperature followed the brain extraction. The tissue was dehydrated using ascending methanol concentrations and incubated in 100% methanol for 2 h at room temperature. Lipids were extracted by incubation in dichloromethane:methanol (2:1) overnight at room temperature and samples were bleached using 5% H_2_O_2_ in methanol overnight at 4 °C. Rehydration was performed by descending methanol concentrations and samples were kept for 24 h at 37 °C in PBS/0.01% sodium azide. Blocking was performed using PBS supplemented with Triton-X 0.2% and porcine gelatin 0.2% for 24 h at 37 °C. Subsequent incubation with primary antibodies lasted for 10 days at 37 °C in the blocking solution. Here primary antibodies included antibodies detecting smooth muscle cells by using anti-αSMA directly coupled to Cy3 (Millipore, cat. no. C6198, 3.75 µg ml^−1^) and antibodies detecting endothelial cells by using anti-podocalyxin (R&D systems, cat. no. MAB1556, 0.5 µg ml^−1^) and anti-CD31 (R&D systems, cat. no. AF3628, 0.133 µg ml^−1^). After washing overnight, secondary antibodies were then incubated for another 7 days at 37 °C. For comprehensive staining of all endothelial cells in the brain, both primary antibodies were combined and visualized by secondary antibodies carrying the same fluorophore. We used anti-goat and anti-rat secondary antibodies coupled to Alexa 647 (Invitrogen, cat. no. A21447, 2.5 µg ml^−1^ and Abcam, ab150155, 2.5 µg ml^−1^, respectively). SMA staining did not include a secondary antibody since the primary antibody is directly coupled to Cy3. After staining, the brains were cleared starting with another dehydration and washing in a dichloromethane/methanol mixture as described above. Benzyl ether was used to optically clear and then transfer to ECi for storage and imaging at room temperature.

### Tissue clearing and sample mounting (zebrafish)

Hatched *Tg(kdrl:GFP)* zebrafish at 3 dpf were anesthetized using 0.02% MS222 and fixed in 4% paraformaldehyde in PBS overnight followed by several washes in PBS. For clearing of the zebrafish larvae, we used an adjusted version of the EZ Clear protocol^20^. The specimens were delipidated in EZ Clear solution (50% tetrahydrofuran in Milli-Q water) for 2 h at room temperature, followed by washes in Milli-Q. RI matching was performed with an adjusted EZ View solution. As reported in the original protocol, the RI of the EZ View solution can be tuned by using different concentrations of urea. Starting at 1.2 M urea, the RI of the EZ View solution was monitored using a digital refractometer (Krüss cat. no. DR301-95; A. Krüss Optronic GmbH) while small amounts of urea were added and dissolved until an RI of *n*_D_ = 1.473 (refractive index of glass capillary) was reached (if the RI is too high, it can be decreased by adding 0.2× PBS). This RI was chosen because it allowed for the convenient mounting of delicate samples and immersion as follows: samples were transferred to a gel consisting of 1.5% low-gelling point agarose in the RI-tuned EZ View and drawn into borosilicate glass capillaries (*n*_D_ = 1.473; ID = 1.0 mm, OD = 1.5 mm, BRAND, cat. no. 701904). The RI of the gel was confirmed before mounting and adjusted by adding Milli-Q if necessary. After gelation of the EZ View agarose, the capillaries were sealed at the bottom with plasticine. Because of the identical RI, glycerol was used as immersion medium as it is inexpensive and does not dry out to form a sticky film and is easier to clean than EZ View.

### Reporting summary

Further information on research design is available in the Nature Portfolio Reporting Summary linked to this article.

## Online content

Any methods, additional references, Nature Portfolio reporting summaries, source data, extended data, supplementary information, acknowledgements, peer review information; details of author contributions and competing interests; and statements of data and code availability are available at https://doi.org/10.1038/s41587-025-02882-8.

## Supplementary information

Supplementary Information Supplementary Notes 1–4 (Resolution and depth of field; vasculature segmentation; mitigating aberrations; voice coil calibration), Figs. 1–8 and Tables 1 and 2.

Reporting Summary

Supplementary Video 1 Concept of sweeping the light sheet across the field of view

Supplementary Video 2 Recorded swept beam with rolling shutter across the field of view

Supplementary Video 3 Isotropic 3D imaging of a cleared mouse cochlea and extracted spiral ganglion neurons (SGNs)

Supplementary Video 4 Isotropic 3D imaging of a cleared zebrafish

Supplementary Video 5 Multicolor isotropic 3D imaging of a cleared mouse cochlea

Supplementary Video 6 Multi-resolution 3D imaging of a cleared mouse brain

## Data Availability

Due to the very large size of the imaging datasets generated in this study, a downsampled version of the complete stitched dataset, together with a representative high-resolution subset, is available via Zenodo at https://doi.org/10.5281/zenodo.17080861(ref. ^43^). The full original high-resolution dataset is available from the corresponding author upon reasonable request. The CAD models of the custom parts are available via Zenodo at https://doi.org/10.5281/zenodo.17396943 (ref. ^44^).

## Code availability

The Python script for the voice coil waveform corrector is available via GitHub at https://github.com/aakhtemostafa/Voice-Coil-Waveform-Correction.git (ref. ^45^).
