# Supplementary material for: Isotropic, aberration-corrected light sheet microscopy for rapid high-resolution imaging of cleared tissue
Source: Nat Biotechnol. 2025 Nov 13;44(11):1858–68. doi: 10.1038/s41587-025-02882-8 (PMC13751298; doi:10.1038/s41587-025-02882-8)
Supplement: Supplementary file 2 [file 41587_2025_2882_MOESM2_ESM.pdf]

nature portfolio

Corresponding author(s): Huisken, Jan

Last updated by author(s): Sep 10, 2025

# Reporting Summary

Nature Portfolio wishes to improve the reproducibility of the work that we publish. This form provides structure for consistency and transparency in reporting. For further information on Nature Portfolio policies, see our Editorial Policies and the Editorial Policy Checklist.

## Statistics

For all statistical analyses, confirm that the following items are present in the figure legend, table legend, main text, or Methods section.

| n/a | Confirmed | |
|---|---|---|
| ☐ | ☒ | The exact sample size (*n*) for each experimental group/condition, given as a discrete number and unit of measurement |
| ☒ | ☐ | A statement on whether measurements were taken from distinct samples or whether the same sample was measured repeatedly |
| ☒ | ☐ | The statistical test(s) used AND whether they are one- or two-sided<br>*Only common tests should be described solely by name; describe more complex techniques in the Methods section.* |
| ☒ | ☐ | A description of all covariates tested |
| ☒ | ☐ | A description of any assumptions or corrections, such as tests of normality and adjustment for multiple comparisons |
| ☐ | ☒ | A full description of the statistical parameters including central tendency (e.g. means) or other basic estimates (e.g. regression coefficient) AND variation (e.g. standard deviation) or associated estimates of uncertainty (e.g. confidence intervals) |
| ☒ | ☐ | For null hypothesis testing, the test statistic (e.g. *F*, *t*, *r*) with confidence intervals, effect sizes, degrees of freedom and *P* value noted<br>*Give P values as exact values whenever suitable.* |
| ☒ | ☐ | For Bayesian analysis, information on the choice of priors and Markov chain Monte Carlo settings |
| ☒ | ☐ | For hierarchical and complex designs, identification of the appropriate level for tests and full reporting of outcomes |
| ☒ | ☐ | Estimates of effect sizes (e.g. Cohen's *d*, Pearson's *r*), indicating how they were calculated |

*Our web collection on statistics for biologists contains articles on many of the points above.*

## Software and code

Policy information about availability of computer code

| | |
|---|---|
| Data collection | The custom codes developed in this study have been deposited in the public repository GitHub (https://github.com/aakhtemostafa/Voice-Coil-Waveform-Correction.git). |
| Data analysis | Data and image processing, and visualization were performed using open-source and commercial software. Initial image analysis was conducted using Fiji, while stitching and conversion to HDF5 were carried out with BigStitcher. 3D data visualization was performed using Imaris 9.7. Point spread funtion measurements were obtained using a custom Python script and Fiji, and the resolution calculations for the detection objective lens were performed in MATLAB. The scripts are available from the authors upon request. |

For manuscripts utilizing custom algorithms or software that are central to the research but not yet described in published literature, software must be made available to editors and reviewers. We strongly encourage code deposition in a community repository (e.g. GitHub). See the Nature Portfolio guidelines for submitting code & software for further information.

## Data

Policy information about availability of data

All manuscripts must include a data availability statement. This statement should provide the following information, where applicable:

- Accession codes, unique identifiers, or web links for publicly available datasets
- A description of any restrictions on data availability
- For clinical datasets or third party data, please ensure that the statement adheres to our policy

Due to the very large size of the imaging datasets generated in this study, a down-sampled version of the complete stitched dataset together with a representative high-resolution subset has been deposited in Zenodo (https://doi.org/10.5281/zenodo.17080861). The full original high-resolution dataset is available from the corresponding author upon reasonable request.
The CAD models of the custom parts are available at the following link: https://doi.org/10.5281/zenodo.17396943

## Research involving human participants, their data, or biological material

Policy information about studies with human participants or human data. See also policy information about sex, gender (identity/presentation), and sexual orientation and race, ethnicity and racism.

| | |
|---|---|
| Reporting on sex and gender | *Use the terms sex (biological attribute) and gender (shaped by social and cultural circumstances) carefully in order to avoid confusing both terms. Indicate if findings apply to only one sex or gender; describe whether sex and gender were considered in study design; whether sex and/or gender was determined based on self-reporting or assigned and methods used. Provide in the source data disaggregated sex and gender data, where this information has been collected, and if consent has been obtained for sharing of individual-level data; provide overall numbers in this Reporting Summary. Please state if this information has not been collected. Report sex- and gender-based analyses where performed, justify reasons for lack of sex- and gender-based analysis.* |
| Reporting on race, ethnicity, or other socially relevant groupings | *Please specify the socially constructed or socially relevant categorization variable(s) used in your manuscript and explain why they were used. Please note that such variables should not be used as proxies for other socially constructed/relevant variables (for example, race or ethnicity should not be used as a proxy for socioeconomic status). Provide clear definitions of the relevant terms used, how they were provided (by the participants/respondents, the researchers, or third parties), and the method(s) used to classify people into the different categories (e.g. self-report, census or administrative data, social media data, etc.) Please provide details about how you controlled for confounding variables in your analyses.* |
| Population characteristics | *Describe the covariate-relevant population characteristics of the human research participants (e.g. age, genotypic information, past and current diagnosis and treatment categories). If you filled out the behavioural & social sciences study design questions and have nothing to add here, write "See above."* |
| Recruitment | *Describe how participants were recruited. Outline any potential self-selection bias or other biases that may be present and how these are likely to impact results.* |
| Ethics oversight | *Identify the organization(s) that approved the study protocol.* |

Note that full information on the approval of the study protocol must also be provided in the manuscript.

# Field-specific reporting

Please select the one below that is the best fit for your research. If you are not sure, read the appropriate sections before making your selection.

☒ Life sciences ☐ Behavioural & social sciences ☐ Ecological, evolutionary & environmental sciences

For a reference copy of the document with all sections, see nature.com/documents/nr-reporting-summary-flat.pdf

# Life sciences study design

All studies must disclose on these points even when the disclosure is negative.

| | |
|---|---|
| Sample size | We did not calculate sample sizes beforehand. To ensure reliable estimation of PSF parameters across the field of view (FOV), we measured the full width at half maximum (FWHM) of seven nano-gold beads per FOV section, resulting in more than 300 beads in total in ASLM mode with and without the concave mirror. The beads were embedded in cleared, refractive-index-matched agarose within the chamber medium.<br><br>For the mouse brain, cochlea, and zebrafish experiments, we did not use a statistical method to determine the number of samples. Instead, we imaged as many samples as were sufficient to demonstrate reproducibility and validate our imaging approach. |
| Data exclusions | No data was excluded. |
| Replication | Replication of the experiment on all imaged samples was successful. |

| | |
|---|---|
| Randomization | The PSF measurements were taken randomly across the large field of view. |
| Blinding | Not applicable. The manuscript describes a new imaging method. |

# Reporting for specific materials, systems and methods

We require information from authors about some types of materials, experimental systems and methods used in many studies. Here, indicate whether each material, system or method listed is relevant to your study. If you are not sure if a list item applies to your research, read the appropriate section before selecting a response.

## Materials & experimental systems

| n/a | Involved in the study | |
|---|---|---|
| ☐ | ☒ | Antibodies |
| ☒ | ☐ | Eukaryotic cell lines |
| ☒ | ☐ | Palaeontology and archaeology |
| ☐ | ☒ | Animals and other organisms |
| ☒ | ☐ | Clinical data |
| ☒ | ☐ | Dual use research of concern |
| ☒ | ☐ | Plants |

## Methods

| n/a | Involved in the study | |
|---|---|---|
| ☒ | ☐ | ChIP-seq |
| ☒ | ☐ | Flow cytometry |
| ☒ | ☐ | MRI-based neuroimaging |

## Antibodies

**Antibodies used**

Mouse Cochlea:
Guinea pig polyclonal anti-parvalbumin antibody (Synaptic Systems, Cat.: 195 004; Lot # 3-38; Clone not specified) – dilution: 1:300
Camelid single domain anti-guinea pig antibody (NanoTag Biotechnologies, Cat.: N0602; Lot # 022205; Clone not specified) labelled with Atto565 – pre-conjugated with 195 004 (Synaptic Systems, see before) at a molar ratio of 1:3.

Multicolor imaging of mouse cochlea: guinea pig anti-parvalbumin (Synaptic Systems, Cat.: 195 004) and the camelid single domain anti-guinea pig antibody (NanoTag Biotechnologies, Cat.: N0602) were used as described before. Additionally, a monoclonal mouse anti-neurofilament 200 antibody (Sigma-Aldrich, Cat.: N5389; Lot # 099M4843V; Clone NE14) at a dilution of 1:400 and a polyclonal rabbit anti-vesicular glutamate transporter 3 antibody (Synaptic Systems, Cat.: 135 203; Lot # 1-47; Clone not specified) at a dilution of 1:300 were used..For the corresponding secondary staining staining an anti-mouse antibody labelled with Alexa 647 (Invitrogen, Cat.: A-21236; Lot # 751096; Clone not specified) and an anti-rabbit antibody labelled with Alexa 488 (Invitrogen; Cat.:, A-11008; # 659082; Clone not specified),were used, each at a dilution of 1:200..

Mouse brain:
Anti-α smooth muscle actin (αSMA) – Directly coupled to Cy3 (Millipore, C6198, 3.75 µg/ml, clone 1A4) - Used for detecting smooth muscle cells (validation: original publication: Skalli, O., et al. A monoclonal antibody against alpha-smooth muscle actin: a new probe for smooth muscle differentiation. J Cell Biol 103, 2787-2796 (1986).)
Anti-podocalyxin (R&D Systems, MAB1556, 0.5 µg/ml, clone 192703) - Used for detecting endothelial cells (validation: Monoclonal antibody used by numerous studies according to manufacturer, validated by the staining pattern)
Anti-CD31 (R&D Systems, AF3628, 0.133 µg/ml, lot not recorded) - Used for detecting endothelial cells (validation: Polyclonal antibody used by numerous studies according to manufacturer, validated by the staining pattern)
Anti-goat secondary antibody coupled to Alexa647 (Invitrogen, A21447, 2.5 µg/ml, lot: 2297623)
Anti-rat secondary antibody coupled to Alexa647 (Abcam, ab150155, 2.5 µg/ml, lot: 2465077)

**Validation**

The usage of all primary antibodies is described in the Methods section for both mouse cochlea and mouse brain preparations.
• Mouse cochlea:
Used for immunofluorescence staining in mouse cochlear tissue.
Duque Afsonso CJ: Development and Application of Tools for the Characterization of the Optogenetics Stimulation of the Cochlea. PhD Thesis. 2020; http://dx.doi.org/10.53846/goediss-8106

• Mouse brain:
• anti-αSMA: Used for immunofluorescence staining of smooth muscle actin in mouse brain vasculature; validated by Skalli, O., et al. A monoclonal antibody against alpha-smooth muscle actin: a new probe for smooth muscle differentiation. J Cell Biol 103, 2787–2796 (1986).
• anti-CD31: Used for immunofluorescence staining of endothelial cells in mouse brain tissue; validated by Western blotting according to manufacturer and by staining pattern; widely used in published studies.
• anti-podocalyxin: Used for immunofluorescence staining of vascular structures in mouse brain tissue; validated by immunocytochemistry according to manufacturer and by staining pattern; widely used in published studies.

## Animals and other research organisms

Policy information about studies involving animals; ARRIVE guidelines recommended for reporting animal research, and Sex and Gender in Research

| | |
|---|---|
| Laboratory animals | Mouse cochlea: Mus musculus, C57BL/6J. The mice were kept at a constant temperature of 22-24°C and at a relative humidity level of 45 to 65% on a 12-hour light/ dark cycle and were provided with enrichment, and access to food and water ad libitum. Mice for extraction of cochleae were bred at the local animal facility of the University Medical Center Göttingen. Here, we used C57BL/6J wild-type mice of both sexes of 1-2 months.<br><br>Mouse Brain: C57BL/6N mice (bred in the animal facility of the University of Lübeck). The mice were kept at a constant temperature (22°C) and humidity (50%) on a 12-hour light/ dark cycle and were provided with standard laboratory chow and water ad libitum. Mice for brain extraction were bred at the local animal facility of the University of Lübeck. Male C57BL6/N mice at the age of 4-6 months were used.<br><br>Zebrafish: transgenic line Tg(kdrl:GFP), 3 dpf |
| Wild animals | / |
| Reporting on sex | Mouse Cochlea: Animals of both sexes were used.<br>Mouse Brain: both sexes weres used for establishing stainings and clearing, no difference was observed, images in the manuscript originate from male mice<br>Zebrafish: The sex cannot be determined at this early stage of development. |
| Field-collected samples | / |
| Ethics oversight | In Germany organ extraction is operated under §4 of the animal wellfare act and not requiring specific permission of the responsbile authorities. Following internal regulations, approval for extraction of cochleae was obtained from the animal welfare office of the University Medical Center (UMG) Göttingen. At University Medical Center Göttingen animals for this study were strictly sacrificed for organ collection only. Thus, a license by the authorities was not required. It was performed according to the regulations of the internal animal welfare office. Experimental procedures were approved by the local animal ethics committee (Ministerium für Landwirtschaft, Umwelt und ländliche Räume, Kiel, Germany).<br><br>Adult zebrafish (Danio rerio), embryos and larvae were maintained following established protocols in compliance with EU directive 2010/63/EU and the German Animal Welfare Act and approved by the authorities of Lower Saxony (Niedersächsisches Landesamt für Verbraucherschutz und Lebensmittelsicherheit, Oldenburg, Germany). |

Note that full information on the approval of the study protocol must also be provided in the manuscript.

## Plants

| | |
|---|---|
| Seed stocks | *Report on the source of all seed stocks or other plant material used. If applicable, state the seed stock centre and catalogue number. If plant specimens were collected from the field, describe the collection location, date and sampling procedures.* |
| Novel plant genotypes | *Describe the methods by which all novel plant genotypes were produced. This includes those generated by transgenic approaches, gene editing, chemical/radiation-based mutagenesis and hybridization. For transgenic lines, describe the transformation method, the number of independent lines analyzed and the generation upon which experiments were performed. For gene-edited lines, describe the editor used, the endogenous sequence targeted for editing, the targeting guide RNA sequence (if applicable) and how the editor was applied.* |
| Authentication | *Describe any authentication procedures for each seed stock used or novel genotype generated. Describe any experiments used to assess the effect of a mutation and, where applicable, how potential secondary effects (e.g. second site T-DNA insertions, mosiacism, off-target gene editing) were examined.* |
